# “Éden Fluminense:” from a reforestation area on the Tijuca massif to a Europeanized public space in Rio de Janeiro in the latter half of the nineteenth century

**DOI:** 10.1590/S0104-59702024000100039en

**Published:** 2024-10-11

**Authors:** Gabriel Paes da Silva Sales, Mariana Reis de Brito, Rejan R. Guedes-Bruni

**Affiliations:** i Professor, Department of Biology/Pontifícia Universidade Católica do Rio de Janeiro. Rio de Janeiro – RJ – Brazil gabrielsales@puc-rio.br; ii Professor, Department of Biology/Pontifícia Universidade Católica do Rio de Janeiro. Rio de Janeiro – RJ – Brazil marianareis2002@puc-rio.br; iii Professor, Department of Biology/Pontifícia Universidade Católica do Rio de Janeiro. Rio de Janeiro – RJ – Brazil rejanbruni@puc-rio.br

**Keywords:** Environmental history, Representations of nature, Public parks, Landscape transformation, Tijuca Forest

## Abstract

This article reflects on the resignification of the Tijuca massif during the latter half of the nineteenth century after the planting of the Tijuca Forest, based on notions of the social uses of forests which were in line with Eurocentric imagery prizing nature. We utilized primary sources from the Brazilian Arquivo Nacional and Biblioteca Nacional, especially the Hemeroteca Digital online collection. The perception of these green areas shifted, with planted forests evolving from solely spaces for future forestry use to also serve as forest parks for recreation and contemplating nature, while still permitting appreciation of fine wood and landscapes and evoking the idea of nationalism and modernity.

Although the forest that blankets the Tijuca massif in the city of Rio de Janeiro currently appears relatively pristine, the composition of its flora and structure of its forest reveal its various uses over time and allow us to recognize many combined and overlapping elements from the past in the modern landscape. There is a variety of material evidence of past human activities, such as pieces of tiles and pottery in the soil, ruins and remains of old properties, as well as a density and distribution pattern of native plant species that proves a certain degree of human interference, along with the presence of exotic plant species.

The Tijuca massif is a chain of mountains that extends over 95km^2^, and over decades it has played and continues to play an essential role in the lives of those who dwell in Rio de Janeiro, and in the past guided the city’s expansion process (Abreu, 2014). Its vegetation is classified as dense submontane and montane ombrophile forest in different stages of succession, covering the massif from its base at 50 meters above sea level to its highest point, Tijuca Peak, at 1,021 meters (IBGE, 2012).

Most of the Tijuca massif is covered by what is commonly known as Atlantic Forest, composed of a sparse substory of shrubs, the occasional vine, and trees that can reach 40 meters tall. Large-diameter trees are rare and stand out in the landscape, such as *jequitibás* (*Cariniana estrellensis* (Raddi) Kuntze and *Cariniana legalis* (Mart.) Kuntze), Spanish cedars (*Cedrela odorata* L. and *Cedrela fissilis* Vell.), *paineiras* (*Ceiba glaziovii* (Kuntze) K. Schum.), and *canelas* (*Ocotea odorifera* (Vell.) Rohwer., *Ocotea glaziovii* Mez. and *Cryptocarya saligna* Mez.). Today, most of the forests on the massif are protected within Tijuca National Park, which is comprised of four unconnected sectors that together cover roughly four thousand hectares (Sales, Guedes-Bruni, 2023).

Since the sixteenth century, the massif was a source of natural resources (mainly water, lumber, and fuel, namely charcoal and firewood) that supplied the establishment and growth of the city (Dean, 1996; Cabral, 2011; Abreu, 2013). This intrinsic relationship between the city of Rio de Janeiro and its forests reduced them to their current size, now spanning 25% of the city’s territory (MapBiomas Brasil, 2022). During the first half of the nineteenth century, most of the vegetation was cut and burned to establish coffee plantations, as part of a model that was intended to protect the soil (Drummond, 1988; Abreu, 2014). The aggravation of problems supplying water to the city of Rio de Janeiro that dated back to its founding led to the adoption of initiatives to preserve its springs and remaining forests, as well as reforestation^1^ of degraded areas (Drummond, 1988, 1997; Heynemann, 1995; Pádua, 2002; Abreu, 2014).

Converting forests into farmland or to produce wood or energy was common in European countries as they developed during the eighteenth and nineteenth centuries. The impacts of this landscape transformation resulted in efforts dedicated to reforestation, since deforestation was a major public concern in those countries such as Spain and England at the time (Vadell, Miguel, Pemán, 2016). These concerns, along with a Brazilian intelligentsia inspired by European scientism, materialized in the tropical South in activities that used rationality to attain the proclaimed ideals of progress.

In this way, various protective measures were proposed from the early nineteenth century, such as a “decree prohibiting the cutting of trees, wood, firewood, and brush throughout the land surrounding the springs of the Carioca River”^2^ in 1817 (Minuta..., 1817). And especially from the 1840s, activities were planned and implemented to preserve the remaining forests and recover the degraded areas. These initially included experimental planting around the Carioca River and the springs that fed it, since this was the main body of water in the city at that time (Abreu, 2014; Martins, 2015; Capilé, 2018; Sales, 2021). The city expanded along with its population, which intensified the need for more forest resources and further impacted its woodlands and made projects to preserve the remaining forests and springs imperative.

The first records of plantings to remediate degraded areas on the Tijuca massif date back to 1843 by Miguel de Frias e Vasconcellos, in the space that was later renamed the Paineiras Forest. During the 1850s, as land was expropriated on different parts of the massif by then imperial minister Luiz Pedreira do Couto Ferraz, the reforestation project was rescaled and in 1861 came to also include the land known as the Tijuca Forest (Bediaga, 2014; Sales, 2021). If these forests had a birth certificate, it would certainly be the ordinance dated December 11, 1861 containing provisional measures on planting and preservation for both (Brasil, 1860-1900). The planting at the Tijuca Forest that began in 1862 was initially led by Manoel Gomes Archer (1862-1874 and 1890-1891) and later Gastão d’Escragnolle (1874-1888), as well as other administrators and interim or short-term leaders such as the French landscape designer Auguste François Marie Glaziou in 1889 (Sales, 2021).

The 1861 ordinance defined regular and systematic planting of the deforested areas that were expropriated in order to be converted into what were then designated the Tijuca and Paineiras Forests, prioritizing the areas adjacent to springs and bodies of water. It also discussed the use of native species (“regular planting of the country’s trees”) and the layout of reforestation in the field. The trees were to be planted in straight, parallel lines, with lines in one direction perpendicular to the others and a distance of 25 palms (5.5m) between saplings. This spacing contained 330 individuals per hectare and 30.25m^2^ for each tree (Brasil, 1860-1900; Sales, Guedes-Bruni, 2023). This distance was intended to allow the saplings to fully develop, easy access to monitor their growth, and the desired spacing between the crowns of the mature trees, which would facilitate future management. At that time, it was acknowledged that these forests could provide a future store of wood that could be harvested for various uses, which determined which species were primarily selected for the planting efforts.

At the same time, from 1850 the process of urban changes intensified in Imperial Brazil through a strong feeling that the evils of the country’s colonial legacy were being overcome and the desire to attain the same levels of development as European nations. The multiple interests that factored into the construction of the Brazilian Empire as a political unit at the time of its political emancipation considered the city of Rio de Janeiro the “head of Brazil” (Mattos, 2005).

The transformations that provided the framework for Brazilian society (such as the Paraguayan War, the abolitionist movement, the shift in the center of coffee culture from the Paraíba Valley to western São Paulo, European immigration, and intellectual renewal as values from liberalism and scientism were assimilated) marked the period between 1850 and 1900 (Lemos, 2009). The Agricultural Congress held in the city of Rio de Janeiro in 1878, for instance, illustrates the political environment of the time in detail (Carvalho, 1988).

During the second half of the nineteenth century, the political centrality of Rio de Janeiro represented tensions between the ideal of incorporating European cultural practices and showcasing its tropical nature (Neves, 2009). This nature, with its terrestrial magnitude (which makes it impossible to consider a single unified nature, as demonstrated by Martius) and potential for economic exploitation, permitted greater dissemination of illuminist and romantic culture (Pádua, 2009).

Taking inspiration from the French capital’s shining model of an urban environment integrated with green spaces, a metropolitan assembly of gardens, plazas, and parks was established across Brazil, along with tree-planting programs (Dourado, 2011). But to understand the growth in green areas that were part of the urban footprint and embodied the habits of the Brazilian population during the mid-eighteen hundreds, we cannot ignore the cultural movement that began to gain strength in western culture between the late eighteenth century and throughout the nineteenth century, as the topic of nature increasingly included and attracted the thought of intellectuals and romantic artists. For this, we need to go back in time, and begin more precisely in eighteenth-century England, which was when the guiding principles of the new romantic cosmology were implemented into the art of establishing gardens (Girons, 2001).

Since the eighteenth century, in England green spaces were recommended as spaces where recreation, leisure, gardening, and contemplating nature would stimulate self-esteem and affirm the dignity of its citizens. Trees planted and arranged to imitate the “wild physiognomy of the forest” led new sensibilities and esthetic values to emerge, but did not minimize the contradictions of modern society with regard to the centrality of humans and their domination of nature (Thomas, 1989). These principles became increasingly important as the world became more industrialized. The representativity of a new working class and broad urban development determined the establishment of a new modern concept of the city that included establishing extensive green areas at the heart of the great metropolises. English policymakers soon understood that a leisure space in nature was welcoming and could provide the strength needed to transform an unlearned population of workers in accordance with the standards of family morality. Within these new urban dimensions, experiments with landscape could take different forms; bucolic experiences were possible, alongside a sense of curiosity about the natural elements. The new English cosmology of landscape construction gradually spread and inspired other regions in Europe and around the world (Dourado, 2011).

The modern concept of the urban was defined at the start of the nineteenth century, as gardens and parks appeared in cities in England and France, most notably parks and public forests constructed near city centers like Regent’s Park in London and the Forest of Fontainebleau southeast of Paris. These green spaces were responsible for encouraging greater proximity to the environment, and promoted a new dialog between civilization and nature that was linked to a sense of fascination with forests (Duarte, 2005). In this way, while the way humans interacted with the biological and physical environment visibly transformed throughout the eighteen hundreds, their relationship with the plant world became increasingly complex as part of the scientific advances that spread throughout the west. These aspects indicate a path towards valuing plants and, in turn, the natural world.

Napoleon III was exposed to the esthetics of English landscape gardens during his exile there, and it was specifically this plastic syntax that inspired the construction of new parks in Paris. Between 1853 and 1870, the city underwent an ambitious transformation led by Baron Haussman: never before had a city received so many parks, gardens, and plazas as Paris during the Second Empire, in just 17 years. Over six hundred thousand trees were planted and no less than 1,835 hectares of green spaces were constructed for the Parisians, who enthusiastically hurried to enjoy these new leisure spaces (Moncan, 2009). While the proliferation of gardens, plazas, and parks made Paris one of Europe’s most beautiful capital cities, it also helped fill the needs of a flourishing industrial bourgeoisie that longed for more green spaces and fresh air, where they could enjoy the typical pursuits of a rising aristocracy like promenading and showing themselves off (Dourado, 2011).

In Brazil, Emperor Dom Pedro II enthusiastically accompanied the transformations underway in Paris (Mérian, 2009), and thanks to the important work of the French landscape designer Auguste François Marie Glaziou he was able during his reign to reproduce the culture of Parisian gardens and the pleasure of contact with green space for Rio’s inhabitants. This shift in social sensibility, associated with ideas of purity and a romantic appropriation of nature, had a strong influence on the cultural value that the forests of the Tijuca massif began to take on, not only for the city of Rio de Janeiro but also for all of Brazil during the second half of the nineteenth century. A public forest in the colonial city was in line with the European “scenario,” reproducing European notions of “prosperity” and “modernity” while also manifesting the emergence of a new ideology that imposed its values by appropriating and manipulating its landscapes (Macedo, 2012). Reforestation of the Tijuca massif, along with Glaziou’s artistic interventions, shared the aspirations that a century earlier guided the creation of large urban parks in England and Haussman’s expansive green plan in France.

In this way, the concept of a city that integrated forest environments into its very fabric required mediation by public authorities. The policies on reforesting the Tijuca massif and its transformation into an urban park, together with the creation of points of reference entrusted to artists, expressed the concerns of the imperial elite and fed the western imagination, ushering in a new form of understanding and experiencing public space. This evokes the idea of cultural landscape, in which the perception of the biological and physical environment encompasses a much more current perspective: a dynamic system of mutual relationships and interferences spanning cultural, economic, ethical, natural, political, and social aspects. In 2009, the Brazilian National Institute of Historic and Artistic Heritage (Iphan) recognized Brazil’s cultural landscape to mean the “specific portion of the national territory that represents the process of man’s interaction with the natural environment, in which life and human science have left marks or attributed values” (Iphan, 2009).

As for Brazilian historiography and the variety of environmental topics it explores (most notably efforts to analyze cultural dimensions of the relationship between humans, nature, and society), there are many approaches ranging from forests, agriculture and ranching, biodiversity and the use of biological resources, as well as spatiotemporal dynamics within the urban context, social diversity, and environmentalism (Pádua, Carvalho, 2020). In this sense, we should also emphasize that environmental history emerged as a revisional effort to enrich history overall in its narratives, utilizing other documentary sources than those traditionally used, and ultimately rejected the otherwise conventional premise that human activity would not lead to ecological consequences for nature on various scales (Worster, 1991; Cronon, 1993; Pádua, 2010).

From the perspective of constructing a narrative on the cultural dimensions of the relationship between society and nature throughout history, here our starting point was the analysis and interpretation of primary documentary sources, written and iconographic sources, as well as artistic expressions featuring flora, nature, forests, and landscapes (Lara, 2008).

In this present study, the Tijuca massif, more specifically the process of spatial transmutation that occurred during the second half of the nineteenth century, is an object of reflection in our attempt to understand it as a constructed landscape resulting from human agency and art which were present in a given time and space and contained their own ideologies, symbolisms, and values. In order to better reflect the syntax of this green space, it is necessary to understand the history of the planting of the Tijuca Forest and the evolution of a new esthetic of representing nature that reverberated with novel cultural habits that led to a new relationship between humans and nature. Our goal is to develop a narrative about the resignification of the Tijuca massif during the latter half of the nineteenth century from the planned social uses of the forest, in line with Eurocentric imagery related to valuing nature.

## “Picturesque trips and restorative picnics:” the Tijuca massif as a dreamlike space

The water crisis in the city of Rio de Janeiro was not a new phenomenon in the nineteenth century, but as it worsened it became one of the main (although not the only) justifications for planting the Tijuca Forest. We can see by the species chosen and the way the forest was established that it was also seen as an opportunity to generate timber resources for future use, staggered for utilization in the short, medium, and long terms (Sales, 2021). As time passed, the resulting green spaces and even the planted trees appear to have acquired other unplanned meanings and purposes. While originally certain species were considered because of the quality of wood they produce for a future harvest, we can see that later the standing trees were also admired and contemplated for their size and flowers, for example.

In Brazil, attention to and praise for local nature, lush and full of potential, represented a path to solidifying the modern concept of the nation. The symbolic construction of the country through valuing the tropical forests as treasures came to represent civilization and progress, approved and encouraged by the entrepreneurs, intellectuals, politicians, and decision makers of the time (Candido, 2004; Perrotta, 2015; Capilé, França, Sales, 2021). This concept was also in line with European practices already underway since the sixteenth and seventeenth centuries calling for city dwellers to travel to experience the coolness of other areas in the summer and the health benefits of mineral or thermal springs as well as the seaside (Perrotta, 2015).

Joaquim Manuel de Macedo (2005), a physician and Brazilian romantic writer who also wrote for the *Jornal do Commercio* newspaper from 1862 to 1863, expresses these values in his columns which were compiled in a book entitled *Um passeio pela cidade do Rio de Janeiro* [A trip through the city of Rio de Janeiro]. His novels such as *A moreninha* and *O moço loiro* depict urban romances and bourgeois customs (Bezerra, 2018). He describes the transforming city, detailing not only the physical scenario but also its immaterial and sensory aspects. As an observer of the city and its customs within a global context, in 1862 in one of his articles Macedo condemns Rio dwellers who preferred to travel to Europe rather than explore their own city. Equally critical are articles from the 1920 in the Brazilian Society of Tourism’s journal that highlight the monotony of Europa instead of the marvelous outings the city of Rio de Janeiro could provide (Perrotta, 2015).

Dom Pedro II played an important role in the urban improvement of the city, which led more foreigners to reside there. This resulted in the first guides “for foreigners” and “for visitors,” even if traveling tourists were still rare. The first Brazilian tour guide from 1862 was entitled *Viagem pitoresca a Petrópolis para servir de roteiro aos viajantes e recordação deste ameno torrão brasileiro* [A picturesque trip to Petrópolis as a route for travelers and recollection of this amenable Brazilian place]. It includes expressions like healthful residence and well-being, heaven on earth, and eternally green Switzerland, for example. Only in 1873 was the first guide about Rio de Janeiro published, by Felix Ferreira, to “clearly and precisely indicate the districts and most notable establishments of this city.” That same year, the Garnier publishing house released the first book on Rio de Janeiro for foreigners staying in the city, without illustrations or maps, entitled *Guia do estrangeiro no Rio de Janeiro e uma notícia histórica sobre os principais monumentos* [A guide for foreigners for Rio de Janeiro and a historical accounting of its main monuments] (Perrotta, 2015).

The public now wished to visit the lush and most recent “garden in the city,” although the area was difficult to access. In the early nineteenth century, the Tijuca massif could only be reached on foot or with the assistance of equines. A new alternative emerged in the mid-eighteen-hundreds when the area became accessible by horse-drawn cable cars that extended to the lower part of the Alto da Boa Vista hill, and the upper part could be reached by rented horse carts. The *Hand book of Rio de Janeiro* published in 1887 features an ad for the Peres e Cia. car company, which specialized in offering visits to city’s sublime mountain scenery, referring to the place with such phrases as “the Eden of Rio” and “picturesque trips” (Perrotta, 2015).

Considering this language, in this study we searched the periodicals of that time (1860-1900) using the portal of the Hemeroteca Digital [Digital Periodicals Collection] at the Brazilian National Library using the corresponding keywords “Eden Fluminense” and “Passeios Pittorescos,” since these terms recurred in the ads. The number of occurrences for each phrase was grouped by decade, while the publications were individually analyzed to verify which were directly related to the Tijuca massif (Table 1). A total of 28 newspapers and periodicals were analyzed, 18 of which mentioned tours, most notably the *Jornal do Commercio* (RJ) and *Almanak Laemmert: Administrativo, Mercantil e Industrial* (RJ), which had the most ads.

**Table 1:** : Number of occurrences for the keywords “Eden Fluminense” [“Eden of Rio”] and “Passeios Pittorescos” [“Picturesque Trips”] by decade (1850-1899)

**Period**	**“Éden Fluminense”**	**“Passeios Pitorescos”**
1850-1859	0	0
1860-1869	0	2
1870-1879	5	6
1880-1889	70	14
1890-1899	5	32

Source: created by the authors, based on the search conducted via the Brazilian Biblioteca Nacional/ Hemeroteca Digital.

In the documents we analyzed, we noted specifically from the 1870s onward various ads describing “tourism routes” to the Tijuca massif offered by the entrepreneur P.A.F. Peres, generally called “Passeios Pitorescos” and “Piqueniques Restauradores” [“Restorative Picnics”]. The intention was to promote a kind of provincial entertainment, where the tropical forest landscape could be explored and contemplated. But considering their prices, these leisure activities were predominantly directed at the elites of Rio de Janeiro.

**Figure 1:**
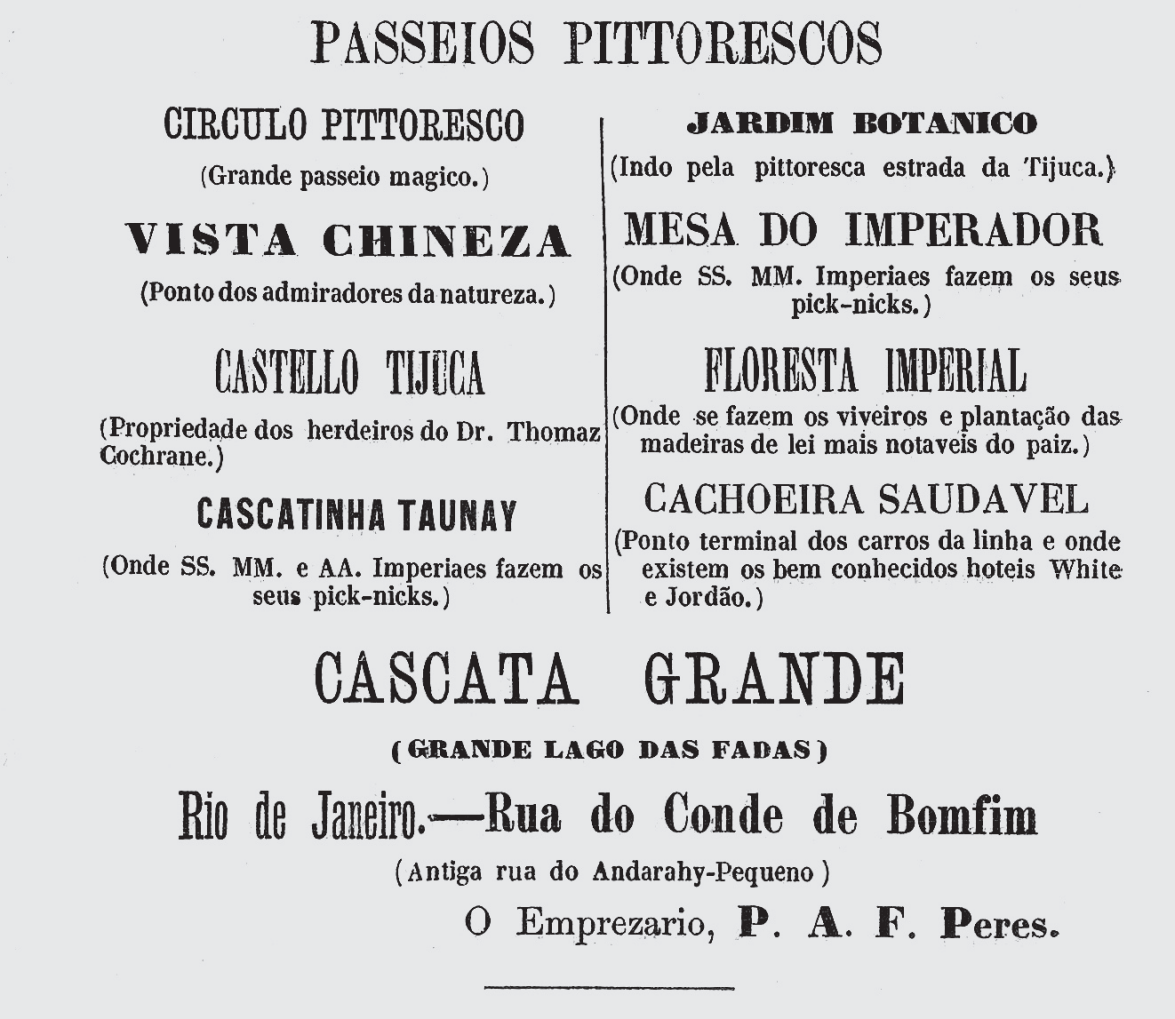
: Advertisement featuring the eight attractions that could be visited on the Tijuca massif (Almanak Administrativo, Mercantil e Industrial do Rio de Janeiro, 1873, p.456)

**Figure 2:**
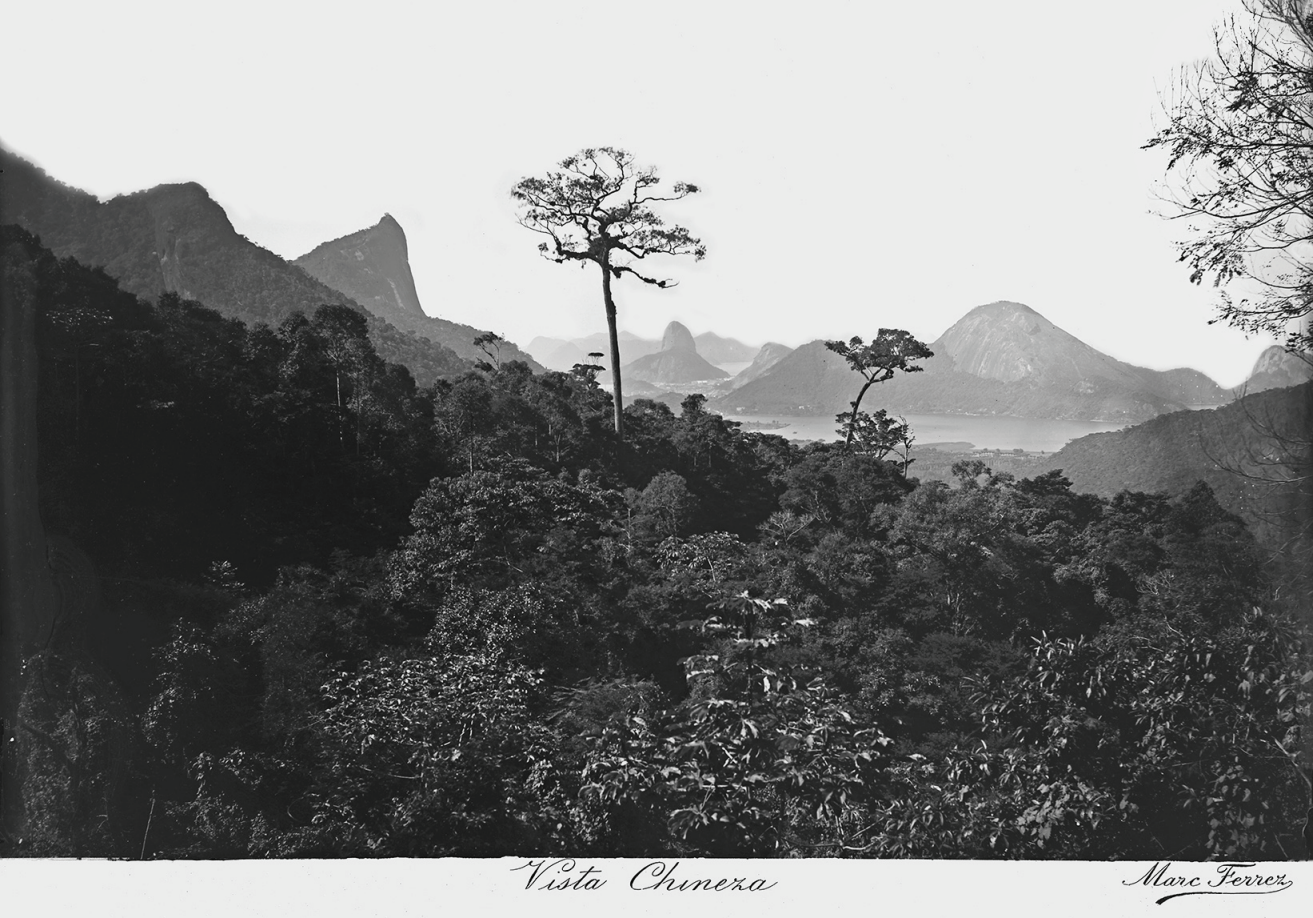
: View of Rio de Janeiro from Vista Chinesa. Photo by Marc Ferrez, c.1885 (Instituto Moreira Salles, Rio de Janeiro)

**Figure 3:**
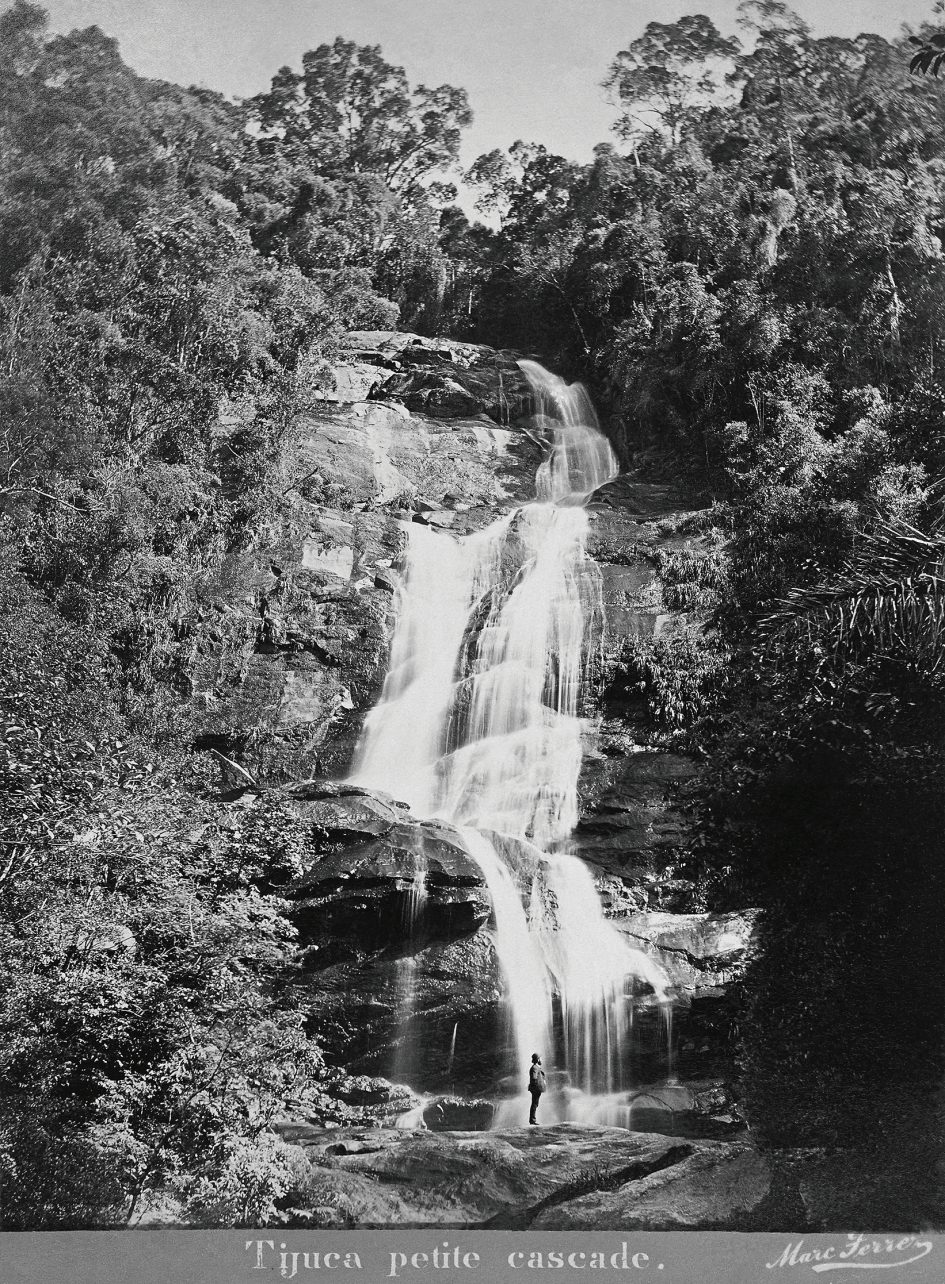
: Cascatinha da Tijuca [Tijuca Falls], also known as Cascatinha Taunay. Photo by Marc Ferrez, c.1885 (Instituto Moreira Salles, Rio de Janeiro)

In 1873, an advertisement published in *Almanak Laemmert: Administrativo, Mercantil e Industrial* (RJ) (Figure 1) advertised cool and enjoyable trips to the Tijuca massif, where depending on the various routes available, visitors could explore the “Círculo Pitoresco” [Picturesque Circle] that provided a “magical grand tour” that allowed them to see such sights as the “Vista Chinesa” [Chinese View], a point for “admirers of nature” (Figure 2), or the “Floresta Imperial” [Imperial Forest] containing “the nurseries and plantings of the country’s most notable wood [species]” and the “Cascatinha Taunay” [Taunay Waterfall], where “Their Imperial Highnesses have their picnics” (Figure 3). That year, other options for enjoying nature involved visiting the “Cachoeira Saudável” [Healthful Waterfall], “Cascata Grande” [Great Falls], “Castelo Tijuca” [Tijuca Castle], “Jardim Botânico” [Botanical Garden], and “Mesa do Imperador” [Emperor’s Table], offering a total of eight attractions. We can also see that the elements of nature so emblematic of the city of Rio de Janeiro were emphasized, from beautiful landscapes to be admired for their various forms, shapes, and colors, or waterfalls and other bodies of water for swimming and cooling off. For example, the Botanical Garden, which originated as an acclimatization garden in 1808, brought visitors into contact with Brazilian flora as well as exotic species (Bediaga, 2007) that could only otherwise be explored on trips abroad.

This paradisiac ideal can be seen in the photo captured by Ferrez (Figure 2) that highlights the rolling terrain of Rio, with the hills of Corcovado (not yet home to the Christ the Redeemer statue) and Pão de Açúcar emerging above, along with the gleaming waters of the Rodrigo de Freitas Lagoon, leaving the viewer to feel as if they were part of the forest. The image of Taunay Falls (Figure 3) emphasizes the grandiose nature of the waterfall and all the forest that surrounds it from the visitor’s perspective, perhaps provoking reflection on the different scales of nature and humanity.

In another advertisement published on January 24, 1874 in *A República*, new attractions were added to the available routes. From this year, tourists purchasing some of these packets could visit other points such as “Alto do Archer” [Archer Peak],^3^ which offered a “botanical view,” “Boa Vista” [Beautiful View], “the site of the grand Victoria Hotel,” “Cascatinha Freitas ou Grupo de Ninfas” [Freitas Falls or the Group of Nymphs], and “Retiro do Ginty” [Ginty’s Retreat]; there were a total of 12 attractions.

As decades passed, considerably more attractions were added to the routes advertised by Peres: from eight in 1873 to 67 in 1890.^4^ Each of these tourist sites had its own qualities, specific reasons that gave them meaning and made them worth visiting.

The expansion of the hotel network beyond the Centro and Catete regions, as well as improved access that extended to Jardim Botânico, Laranjeiras, Gávea, and Tijuca (in the Metrópole, Belvedere, Botanical Recreio de Olaria, Corcovado, and Aurora Hotels, among others) facilitated consumption of this tourism product that prized the vibrant nature of the city of Rio de Janeiro through different routes (Perrotta, 2015).

Only in the 1887 publication *Impressões de viagem Brasil-Europa* are the better-known tourist spots presented, including Corcovado, Paineiras, and Alto da Boa Vista (including Cascata Grande, Cascatinha Pequena, and the Tijuca National Forest). These were associated with tram stations and the availability of hotels nearby (Perrotta, 2015). In this sense, the trams played an important role in the urban expansion of the city, led by the Companhia de Carris de Ferro da Cidade à Boavista na Tijuca, with this company’s line inaugurated in 1859 by the emperor (Von der Weid, 1994).

At the start of the nineteenth century, the ongoing and expanding influx of foreigners in Rio de Janeiro led to greater movement between the provinces, with the city becoming the main destination for visitors who had sufficient time and resources to visit and explore it (Machado, 2013), along with the urban facilities required to make their visits more comfortable.

We should also note that demand for these trips to the green areas of the Tijuca massif were also related to the possibility of experiencing a more agreeable climate. This consequently was an affirmation of certain spaces as hygienic in the midst of a growing city that needed green areas where healthy conditions could be established (Cardozo, 2016).

## The Eden of Rio: the transmutation of the Tijuca massif during the second half of the nineteenth century

Our analysis of advertisements in periodicals of the era demonstrated the symbolic value attributed to the Tijuca massif that was part of the urban fabric of Rio in the mid-eighteen-hundreds. This evokes an interesting horizon in which the emphasis on totality is thought to be one of the most encompassing dimensions of the Romantic movement (Duarte, 2005). However, more profound exploration of this ideological element reveals that totality can often come to mean unity, expressed for example in the consolidation of the modern concept of the nation which took place throughout the nineteenth century through the arduous work of systematically incorporating the treasures present in the country’s territory and its population’s habits and customs, through the intermediary of history. This viewpoint highlights the search for national identity, which was configured according to a new spirit of describing, valuing, and understanding the country’s cultural traditions and natural landscapes.

Unlike the Romantic attitudes that marked the construction of identify in European nations during the nineteenth century, where the customs of rural populations and successive material accomplishments of civilizing processes over time were the main elements of authenticity (Thiesse, 1999), in Brazil uncovering and valuing local wild areas represented a pathway to symbolic nation-building. Literature, poetry, and painting adapted to the desire for differentiation, depicting and praising the unique wealth contained in Brazil’s forests (Candido, 2004).

The widespread exploration of the tropics and discovery of thousands of species previously unknown to the scientific world led to the emergence of a new aesthetic paradigm in Europe that reverberated across the globe: the value and appreciation of landscapes encompassing a vast diversity of plants, spaces for contemplating nature, as well as usefulness in terms of human knowledge about the plant kingdom. From this perspective urban green areas and their social uses are even more important, such as the “picturesque trips” and “restorative picnics” on the Tijuca massif. The creation of carefully planned routes to visit the “Eden of Rio” promoted enchantment by the dense tropical forest, with emotions resulting from the meeting of beauty and the sublime, beliefs that commended green areas.

The collective desire to feel liberty, peace, happiness, alternating with feelings of sadness and melancholy, symbolized a phase of closeness to natural resources through constant *promenades* in the city’s gardens and parks. These were increasingly refined in somber scenes full of drama and in the pursuit of replicating natural, untouched landscapes, establishing links in a new relationship between western humanity and nature (Schama, 1996).

With Brazil’s independence in 1822, the city of Rio de Janeiro became the seat of the Empire, definitively securing its role as the center of the nation and its fundamental function as its intellectual and artistic hub (Candido, 2004). Within this context, nothing would be more intelligent than acclaiming its forests and the proliferation of verdant spaces, places that suggested sophistication and modernity where families should have picnics and appreciate these green areas. They were places that the Brazilian metropolis could be proud of. They were also strongly recommended by health authorities to recover the groundwater and for sunlight and air circulation, thus avoiding the formation of putrid areas that spawned illnesses (Lawrence, 2008) and characterized much of the rest of the city. Revering, exploring, and recognizing the specific characteristics of local vegetation represented a manifestation of prosperity for the tropical capital, signaling greatly desired proximity to the civilized European nations.

This study suggests that the contemplative attitude to Brazil’s abundant and diverse natural resources with their boundless potential was centrally important for the Tijuca massif. The number of attractions in the forest that expanded as the years passed, as seen through newspaper advertisements, can be read from the viewpoint of establishing a memory for the recently-formed nation as a device to make these landscapes and green leisure spaces part of the country’s social and symbolic imagery, thus creating a highly federative attitude.

According to Duarte (2005), as the local nature became a source of pride and this patriotic sentiment spread and became ingrained in the population, it became a fundamental component to strengthen the royal courts. The Tijuca massif experienced this resignification and came to symbolize the glamourous “Eden of Rio” for Brazilians: a rich national treasure, domesticated and accessible, lending credence to a collective consciousness of the importance and authenticity of its unique nature that was adapted to express Brazil’s new singular status as a nation.

## Final considerations

Within the context of the Tijuca massif, the three main administrators of the Tijuca Forest left the marks of their own personalities, along with social demands local politics, and foreign trends linked to the relationship between humans, society, the city, and the natural world. Several attractions on the Tijuca massif were named after these characters: Alto do Major Archer, Ponte do Barão d’Escragnolle, and Refúgio Glaziou, to cite just three examples.

Archer’s pragmatism in reforestation and showcasing the native flora that had been destroyed can be inferred by his preference for Brazil’s native species in his plan, which was primarily intended to protect springs and bodies of water. Among these native species, he also chose good producers of timber as part of a future strategy to generate financial resources for the Empire. The city’s modern forest cover consequently reveals the intentionality of Archer’s original plan without ignoring the natural processes of regeneration from the planted areas.

As a cultured man, d’Escragnolle’s European gaze and artistic sensibilities drove him to beautify the Tijuca Forest with roads, bridges, fountains, and overlooks that were constructed in line with Romantic European notions of the city, thus expanding this space originally established and understood to be a forest protection area into a public park meant for public visitation.

Despite his short administration, Glaziou also integrated his aesthetics of botany and court landscaping into the collection, production, propagation, and planting of aesthetically valuable and eye-catching species. He also used individuals remaining in the better-preserved areas of the massif not only as specimens documented in his herbarium, but also for landscaping in other areas of the city.

The different representations of the artificial landscapes, over time and space, provide with clues that allow us to penetrate the complex relationship between societies and their environment, and to understand the transformation of imagery that guides the value of new meanings and perhaps even a new cognitive geometry of nature.

Exploring the various attractions on the Tijuca massif that were described in newspaper advertisements from the nineteenth century was essential for understanding how the green spaces and natural landscapes located within the urban context of Rio de Janeiro expressed the demands, interests, and expectations of Brazilian society (and more specifically, its aristocratic elites) that marked the historical period in question.

Understanding, valuing, and experiencing the Tijuca massif consequently represented the construction of a federative social memory, and the affirmation of a Brazilian identity that, through its forest treasures and its exuberant nature with myriad qualities and potential, was proud of its nation.

## NOTES

1 Reforestation can be briefly summarized here as restoring forest cover in areas where forests existed previously (Magnanini, 1960). Considering the complexity of this topic and prolific resulting literature, as well as the fact that this article does not address the history of reforestation on the Tijuca massif or earlier environmental policy on this subject, we recommend Rodrigues et al. (2009) for a better understanding of this topic from the viewpoint of ecological restoration, or Moretto (2016) and Ribeiro and Moretto (2022) with regard to the environmental history, for example.

2 In this and other citations of texts from Portuguese, a free translation has been provided.

3 This place name survives to the modern day; it can be seen from Bom Retiro in the Tijuca Forest Sector of Tijuca National Park.

4 An advertisement published in the *Jornal do Commercio* newspaper on December 23, 1890 offered the following attractions: Caixa d’água Aurora, Caixa d’água Frias Vasconcellos, Avenida da Redemptora, Cascatinha Freitas, Praça da Boa Vista, Parque Itamaraty, Castello Leopoldina, Ermida Carvalhaes, Cachoeira Saudável (Posto da Comp. de Educação), Furnas de Agassiz, Grande Cascata Serpa Pinto, Círculo do Barão do Bom Retiro, Cascatinha do Barão de Taunay, Bica do Daniel, Parque Bonfim, Capella do Mesquita, Floresta Nacional, Praça Solon, Boulevard Deodoro, Rua Sampaio Ferraz, Ponte do Barão de Escragnolle, Caminho da Glória, Fonte Marianinha, Pedra Raul, Entrada do Paraíso, Praça Belford Roxo, Excelso, Rua Ruy Barbosa, Recreio Benjamin Constant, Rua Manoel Felizardo, Vista Wandenkolk, Fonte Jesuína, Éden Major Freitas, Alto do Major Archer, Rua Quintino Bocaiúva, Rua Major Campos, Rua Glicério, Praça d’Escragnolle, Praça Quinze de Novembro, Viveiro Florestal, Rua General José Simeão, Descanso Marietta, Cascata Diamantina, Ponte da Baronesa, Gruta de Paulo e Virgínia, Gruta do Constâncio, Abrigo do Chico Christão, Labirinto, Cascatinha Gabriella, Descanso de Dom Pedro de Alcântara, Lago das Fadas, Refúgio Glaziou, Rua Campos Salles, Rua Demétrio Ribeiro, Rua do Açude, Ponte do Chico da Serra, Retiro do Ginty, Parque Lemgruber, Pedra Bonita, Parque Cochrane, Castello Alencar, Recreio Mock, Vista dos Millords, Vista Chinesa, and Jardim Botânico.
