# Supplementary material for: “Éden Fluminense:” from a reforestation area on the Tijuca massif to a Europeanized public space in Rio de Janeiro in the latter half of the nineteenth century
Source: Hist Cienc Saude Manguinhos. 2024 Oct 11;31:e2024039. doi: 10.1590/S0104-59702024000100039en (PMC11472787; doi:10.1590/S0104-59702024000100039en)
Supplement: Translation

# Translation: “Éden Fluminense”: de área de reflorestamento no maciço da Tijuca a espaço público europeizado do Rio de Janeiro na segunda metade do século XIX

**DOI:** 10.1590/S0104-59702024000100039

**Authors:** Gabriel Paes da Silva Sales, Mariana Reis de Brito, Rejan R. Guedes-Bruni

**Affiliations:** i Professor, Departamento de Biologia/Pontifícia Universidade Católica do Rio de Janeiro. Rio de Janeiro – RJ – Brasil gabrielsales@puc-rio.br; ii Professora, Departamento de Biologia/Pontifícia Universidade Católica do Rio de Janeiro. Rio de Janeiro – RJ – Brasil marianareis2002@puc-rio.br; iii Professora, Departamento de Biologia/Pontifícia Universidade Católica do Rio de Janeiro. Rio de Janeiro – RJ – Brasil rejanbruni@puc-rio.br

**Keywords:** História ambiental, Representações da natureza, Parques públicos, Transformação da paisagem, Floresta da Tijuca

## Abstract

O artigo reflexiona sobre o processo de ressignificação do maciço da Tijuca, na segunda metade do século XIX, após o projeto de plantio da Floresta da Tijuca, a partir da proposição de usos sociais das florestas, alinhando-se ao imaginário eurocêntrico de valorização da natureza. Vale-se de fontes primárias do Arquivo Nacional e da Biblioteca Nacional, especialmente da Hemeroteca Digital. Desvela a mudança na percepção dessas áreas verdes, onde a floresta plantada deixou de ser unicamente um espaço para uso silvicultural futuro, passando a ser também uma floresta-parque, para recreação e contemplação da natureza, sem excluir a apreciação das madeiras nobres e das paisagens, evocando a ideia de nacionalismo e modernidade.

O manto florestal que recobre o maciço da Tijuca, na cidade do Rio de Janeiro, apesar de apresentar certa aparência prístina nos dias vigentes, revela, em sua composição florística e estrutura florestal, os diferentes usos que dele foram feitos no decorrer do tempo. Torna-se possível, portanto, reconhecer na paisagem atual, de maneira combinada e sobreposta, numerosos elementos do passado. Os testemunhos materiais das atividades antrópicas pretéritas são diversos, tais como restos de azulejos e de cerâmicas no solo, ruínas e vestígios de antigas propriedades, assim como um padrão de densidade e distribuição de espécies vegetais nativas que evidenciam, em algum grau, a interferência antrópica e a ocorrência de espécies vegetais exóticas, entre outros aspectos.

O maciço da Tijuca constitui-se como uma cadeia de montanhas que se estende por área de 95km^2^ e, ao longo das décadas, exerceu e ainda exerce um papel fundamental na vida dos citadinos, direcionando, no passado, o processo de expansão da cidade (Abreu, 2014). Sua vegetação caracteriza-se como floresta ombrófila densa submontana e montana, em diferentes estágios sucessionais, recobrindo desde o sopé do maciço, a 50msnm, até as proximidades de seu ponto mais alto: o pico da Tijuca, com 1.021msnm (IBGE, 2012).

Popularmente conhecida como Mata Atlântica, em grande parte do maciço da Tijuca, a floresta é constituída por um sub-bosque pouco adensado de arbustos, raras trepadeiras e lianas, cujas árvores podem atingir até 40m de altura. Raros, porém, são os indivíduos arbóreos com grandes diâmetros, e que se destacam na paisagem, como exemplificam os jequitibás (*Cariniana estrellensis* (Raddi) Kuntze e *Cariniana legalis* (Mart.) Kuntze), cedros (*Cedrela odorata* L. e *Cedrela fissilis* Vell.), paineiras (*Ceiba glaziovii* (Kuntze) K. Schum.), canelas (*Ocotea odorifera* (Vell.) Rohwer., *Ocotea glaziovii* Mez. e *Cryptocarya saligna* Mez.), dentre outros. Atualmente, a maior parte das florestas do maciço da Tijuca está protegida pelo Parque Nacional da Tijuca, constituído por quatro setores descontínuos, que somados totalizam cerca de quatro mil hectares (Sales, Guedes-Bruni, 2023).

Desde o século XVI, o maciço foi fonte de recursos naturais (água, carvão, lenha e madeira, principalmente) para provimento ao estabelecimento e crescimento da cidade (Dean, 1996; Cabral, 2011; Abreu, 2013). A intrínseca relação entre a cidade do Rio de Janeiro e suas florestas resultou na redução florestal aos atuais 25% do território carioca (MapBiomas Brasil, 2022). Na primeira metade do século XIX, grande parte da vegetação foi derrubada e queimada para dar lugar à cafeicultura, num modelo que preteriu resguardar o solo (Drummond, 1988; Abreu, 2014). O agravamento da crise de abastecimento hídrico da cidade do Rio de Janeiro, problemático desde a sua fundação, sucedeu na adoção de iniciativas dedicadas à conservação de suas nascentes e dos remanescentes florestais, além do reflorestamento^1^ das áreas devastadas (Drummond, 1988, 1997; Heynemann, 1995; Pádua, 2002; Abreu, 2014).

A conversão de florestas em áreas agrícolas, produtoras de madeira e de energia era prática comum nos países em desenvolvimento na Europa durante os séculos XVIII e XIX. Os impactos decorrentes dessa transformação da paisagem resultaram em ações dedicadas ao reflorestamento, visto que o desmatamento encerrava uma grande preocupação pública naqueles países na época, como exemplificam Espanha e Inglaterra (Vadell, Miguel, Pemán, 2016). Tais preocupações, com uma intelectualidade brasileira inspirada pelo cientificismo europeu, no Sul tropical, materializavam-se em atos que se valiam da racionalidade para atingir os preconizados ideais de progresso.

Dessa maneira, desde o início do século XIX, foram propostas diferentes medidas protetivas, como, por exemplo, o “decreto que proíbe o corte de árvores, madeiras, lenhas e matos, em todo o terreno que rodeie as nascentes do rio Carioca”, em 1817 (Minuta..., 1817). E, a partir, principalmente, da década de 1840, foram concebidas e implementadas ações para a conservação das florestas que ainda estivessem de pé; assim como a recuperação das áreas degradadas que incluíram plantios experimentais, conduzidos, num primeiro momento, nas nascentes e proximidades do rio Carioca, visto tratar-se do principal curso d’água da cidade naquele período (Abreu, 2014; Martins, 2015; Capilé, 2018; Sales, 2021). A expansão da urbe ocorria juntamente com o crescimento de sua população, o que intensificou a necessidade por mais recursos florestais, impactando sobremaneira as matas cariocas. Assim, projetos visando à conservação das florestas remanescentes e das nascentes se tornaram imperiosos.

Os primeiros registros sobre plantios para a recuperação de áreas degradadas no maciço da Tijuca são datados de 1843 e foram conduzidos por Miguel de Frias e Vasconcellos, no território que, posteriormente, foi nomeado Floresta das Paineiras. Na década de 1850, com as desapropriações de terrenos em diferentes partes desse maciço, conduzidas pelo então ministro do Império, Luiz Pedreira do Couto Ferraz, o projeto de reflorestamento foi redimensionado, incluindo também o território determinado, em 1861, como Floresta da Tijuca (Bediaga, 2014; Sales, 2021). Se essas florestas tivessem certidão de nascimento, certamente corresponderia à portaria de 11 de dezembro de 1861, que dispunha sobre as instruções provisórias para o plantio e conservação de ambas (Brasil, 1860-1900). No que se refere especificamente ao plantio da Floresta da Tijuca, iniciado em 1862, foi conduzido por Manoel Gomes Archer (1862-1874 e 1890-1891), seu primeiro responsável e, em um segundo momento, por Gastão d’Escragnolle (1874-1888), além de outros administradores, interinos ou ocupantes do cargo por curtos períodos, como foi o caso do paisagista francês Auguste François Marie Glaziou, em 1889 (Sales, 2021).

Na portaria de 11 de dezembro de 1861 era previsto o plantio regular e sistemático das áreas desmatadas que foram desapropriadas para serem convertidas nas então designadas Floresta da Tijuca e Floresta das Paineiras, prioritariamente nas porções adjacentes às nascentes e ao longo dos cursos d’água. O instrumento orientava também sobre o uso de espécies nativas (“plantação regular de arvoredo do país”), assim como o desenho do reflorestamento em campo. Os plantios deveriam ser feitos em linhas retas, paralelas entre si, sendo as fileiras de uma direção perpendiculares às outras, com distanciamento entre as mudas de 25 palmos, ou seja, 5,5m. Tratava-se, à vista disso, de um plantio que comportaria 330 indivíduos/ha, considerando que o espaço ocupado por muda corresponderia a 30,25m^2^ (Brasil, 1860-1900; Sales, Guedes-Bruni, 2023). Tal distanciamento intentava o pleno desenvolvimento dos indivíduos arbóreos, o fácil acesso para o monitoramento do crescimento das mudas, além de permitir a espacialidade desejada entre as copas das árvores quando maduras, o que facilitaria seu futuro manejo. Naquele momento, vislumbrava-se a ideia predominante de que tais florestas pudessem funcionar como um futuro repositório de madeiras, prevendo seu corte para diferentes finalidades, fato este que orientou a seleção de espécies prioritariamente utilizadas nos plantios.

Paralelamente a tais acontecimentos, intensifica-se, a partir de 1850, o processo de mudanças urbanas no Brasil Império, por meio de um forte sentimento de superação das mazelas da herança colonial e do desejo de alcançar o mesmo estágio de desenvolvimento das nações europeias. Os múltiplos interesses fatoriais na construção da unidade política do Império brasileiro, à época de sua emancipação política, tiveram a cidade do Rio de Janeiro como “cabeça do Brasil” (Mattos, 2005).

Transformações estruturantes na sociedade brasileira – como a guerra do Paraguai, o movimento abolicionista, a mudança do polo cafeicultor do vale do Paraíba para o oeste de São Paulo, a imigração europeia, a renovação intelectual por intermédio da assimilação de valores do liberalismo e do cientificismo, entre outros – marcaram o período de 1850 a 1900 (Lemos, 2009). O Congresso Agrícola, realizado em 1878, na cidade do Rio de Janeiro, por exemplo, ilustra com precisão o ambiente político do período (Carvalho, 1988).

A centralidade política do Rio de Janeiro expressava, na segunda metade do século XIX, a síntese das tensões entre o ideal por incorporar as práticas da cultura europeia, tanto quanto o de desvelar a natureza tropical (Neves, 2009). Natureza cuja magnitude territorial (impeditiva de compreendê-la como natureza única, como desvelado por Martius) e potencialidades de exploração econômica convergiram e facultaram maior difusão das culturas iluminista e romântica (Pádua, 2009).

Buscando inspiração na capital francesa, que irradiava seu modelo de ambiente urbano integrado a espaços verdes, observou-se a implantação de um conjunto metropolitano de jardins, praças e parques, assim como programas de arborização em diversas cidades brasileiras (Dourado, 2011). Entretanto, para compreender o crescimento de áreas verdes que integravam o traçado urbano e modificavam os hábitos da população brasileira, em meados dos Oitocentos, não se pode ignorar o movimento cultural que começou a figurar e ganhar expressividade na cultura ocidental entre o final do século XVIII e o decorrer do XIX, em que a temática da natureza envolvia e atraía, cada vez mais, o pensamento de intelectuais e artistas românticos. Torna-se necessário, portanto, um retrocesso temporal. Iniciando, mais precisamente, na Inglaterra setecentista, que inaugura a inserção dos princípios norteadores da nova cosmologia romântica à arte de execução de jardins (Girons, 2001).

Desde o século XVIII, a Inglaterra incentivava a vivência em espaços verdes, onde as atividades de recreação, descanso, jardinagem e contemplação da natureza seriam um estímulo à autoestima e afirmação da dignidade dos cidadãos. Árvores plantadas e ordenadas, resgatando a fisionomia “selvagem das florestas”, faziam emergir novas sensibilidades e valores estéticos sem, contudo, minimizar as contradições da sociedade moderna no que concerne à centralidade humana e sua dominação da natureza (Thomas, 1989). Esses princípios se tornaram cada vez mais importantes à medida que o mundo se industrializava. A representatividade de uma nova classe trabalhadora e o amplo desenvolvimento urbano determinaram o delineamento de um conceito moderno de cidade, com a implantação de extensas áreas verdes no cerne das grandes metrópoles. Os dirigentes ingleses logo entenderam que um local de lazer, junto à natureza, seria acolhedor e capaz de proporcionar a força necessária para transformar, em padrões de moralidade familiar, uma rude população operária. Nessas novas dimensões urbanas, a paisagem podia ser experimentada de diferentes formas. Era possível degustar a experiência bucólica, atrelada a uma sensação de curiosidade pelos elementos da natureza. A nova cosmologia inglesa de construção da paisagem foi, aos poucos, disseminando-se e inspirando demais regiões da Europa e do mundo (Dourado, 2011).

No início do século XIX, o conceito moderno de urbano foi sendo delineado juntamente com o aparecimento dos jardins e parques em cidades da Inglaterra e da França, sobretudo na construção de parques e florestas públicas, próximas às metrópoles, como o Regent’s Park, para Londres, e a Floresta de Fontainebleau, para Paris. Esses espaços verdes foram responsáveis por estimular mais proximidade com o meio ambiente, promovendo novo diálogo entre civilização e natureza, atrelado a uma sensação de fascínio pelas florestas (Duarte, 2005). Assim, ao longo dos Oitocentos, ao mesmo tempo que se observa uma transformação da interação do ser humano com o ambiente biofísico, a relação com o mundo vegetal foi se tornando cada vez mais complexa, como parte do avanço científico que se ampliava no Ocidente. Esses aspectos apontam para uma via de valorização das plantas e, consequentemente, do mundo natural.

Napoleão III, durante seu exílio na Inglaterra, compreendeu a estética dos jardins paisagísticos ingleses. E foi justamente essa sintaxe plástica que inspirou a construção dos novos parques de Paris. Entre 1853 e 1870, a cidade passou por uma ambiciosa transformação, dirigida pelo barão Haussman. Jamais uma cidade, em apenas 17 anos, recebeu um número tão expressivo de parques, jardins e praças quanto a Paris do Segundo Império. Foram plantadas mais de seiscentas mil árvores e construídos nada menos do que 1.835 hectares de áreas verdes para os parisienses, os quais se apressavam, com entusiasmo, em desfrutar desses novos locais de deleite (Moncan, 2009). A proliferação de jardins, praças e parques, ao mesmo tempo que colocou Paris no rol das capitais mais belas da Europa, contribuiu para atender às demandas de uma florescente burguesia industrial que almejava mais cenários verdes, ao ar livre, onde pudesse exercer comportamentos típicos de uma aristocracia em ascensão, como exibir-se, pavonear-se (Dourado, 2011).

No Brasil, o imperador dom Pedro II acompanhava com interesse e entusiasmo as transformações que aconteciam em Paris (Mérian, 2009) e, graças aos importantes trabalhos paisagísticos do francês Auguste François Marie Glaziou, conseguiu reproduzir em seu império a cultura dos jardins parisienses, que estava atrelada ao prazer dos habitantes da cidade em estar em contato com o verde. Essa mudança de sensibilidade social, associada às ideias de pureza e de apropriação romântica da natureza, desempenhou forte influência no valor cultural que as matas do maciço da Tijuca passaram a representar não apenas para a cidade do Rio de Janeiro, mas, também, para o Brasil, na segunda metade do século XIX. Uma floresta pública na cidade colonial alinhava-se ao “cenário” europeu, imprimindo noções de “prosperidade” e “modernidade”, tanto como manifestava o surgimento de uma nova ideologia que impunha seus valores por meio da apropriação e manipulação das suas paisagens (Macedo, 2012). O reflorestamento do maciço da Tijuca, associado às intervenções artísticas de Glaziou, compartilhava as aspirações que nortearam, um século antes, a criação dos grandes parques urbanos na Inglaterra e o vasto programa verde de Haussman, na França.

Assim, o conceito de cidade integrada a ambientes florestais em seu tecido exigia mediação por parte dos poderes públicos. As políticas de reflorestamento do maciço da Tijuca e a sua transmutação em parque urbano, aliadas à criação de pontos de referência, cuja realização foi confiada a artistas, expressavam os anseios da elite imperial e alimentavam o imaginário ocidental, inaugurando outra forma de conceber e vivenciar o espaço público. Evoca-se, dessa maneira, a ideia de paisagem cultural, na qual a percepção do ambiente biofísico compreende sua perspectiva mais atual: um sistema dinâmico, de relações e interferências mútuas, abrangendo aspectos culturais, econômicos, éticos, naturais, políticos e sociais. Em 2009, o Instituto do Patrimônio Histórico e Artístico Nacional (Iphan) conceituou a expressão paisagem cultural brasileira como a “porção peculiar do território nacional, representativa do processo de interação do homem com o meio natural, à qual a vida e a ciência humana imprimiram marcas, ou atribuíram valores” (Iphan, 2009).

No que concerne à historiografia brasileira e aos variados temas ambientais explorados, sobretudo na proposta de analisar as dimensões culturais da relação ser humano, natureza e sociedade, as abordagens são múltiplas, tratando desde as florestas, agricultura e pecuária, biodiversidade e uso de recursos biológicos, assim como dinâmicas espaçotemporais no contexto urbano, sociodiversidade e ambientalismo (Pádua, Carvalho, 2020). Nesse sentido, cabe destacar, ainda, que a história ambiental surgiu de um esforço revisional para enriquecer a grande área da história em suas narrativas, valendo-se de outras fontes documentais, diferentes daquelas tradicionalmente empregadas, e acabou por rejeitar a premissa, outrora convencional, de que a ação humana não ocasionaria consequências ecológicas à natureza, em diferentes escalas (Worster, 1991; Cronon, 1993; Pádua, 2010).

Na perspectiva da construção de uma narrativa sobre as dimensões culturais da relação entre sociedade e natureza ao longo da história, toma-se como ponto de partida a análise e interpretação de fontes documentais primárias, evidências escritas e iconográficas, tanto como expressões artísticas que vislumbrem a flora, a natureza, as florestas e as paisagens (Lara, 2008).

No presente estudo, o maciço da Tiuca, especificamente o processo de transmutação espacial que ocorreu na segunda metade do século XIX, será o objeto de reflexão na tentativa de o compreender como paisagem construída, resultado da agência e da arte humana, presentes num dado tempo e espaço, carregado de ideologias, simbolismos e valores. A fim de melhor refletir sobre a sintaxe desse espaço verde, torna-se necessário compreender a história do plantio da Floresta da Tijuca e a evolução de uma nova estética de representação da natureza, que reverberava novos hábitos culturais, conduzindo a uma nova relação ser humano/natureza. Objetiva-se, portanto, desenvolver uma narrativa sobre a ressignificação do maciço da Tijuca, na segunda metade dos Oitocentos, a partir da proposição de usos sociais da floresta, alinhando-se ao imaginário eurocêntrico de valorização da natureza.

## “Passeios pitorescos e piqueniques restauradores”: o maciço da Tijuca como um lugar onírico

No decorrer do século XIX, a crise hídrica na cidade do Rio de Janeiro não era novidade, mas seu agravamento foi a principal justificativa para a realização do plantio da Floresta da Tijuca, sem, contudo, limitar-se a tal. Percebe-se, pela seleção das espécies utilizadas e pelo modo como o empreendimento florestal foi conduzido, que a floresta plantada foi concebida como oportunidade também de geração de recursos madeireiros para uso futuro, escalonado, tanto para o curto quanto para médio e longo prazos (Sales, 2021). E mais, no transcorrer dos anos, os espaços verdes produzidos, e até mesmo as árvores plantadas, aparentemente passaram a ter outros significados e finalidades, igualmente não planejadas, daquelas originalmente idealizadas. Se num primeiro momento optou-se por plantar determinada espécie em razão da boa qualidade de sua madeira, prevendo seu corte no futuro, verifica-se que, num segundo momento, a árvore de pé poderia ser admirada e contemplada, devido ao seu porte, floração ou qualidade da madeira, por exemplo.

No Brasil, a atenção e exaltação dispensadas às naturezas locais, exuberantes e repletas de potencialidades representavam um caminho para a consolidação do conceito moderno de nação. A construção simbólica de pátria, edificada a partir do enaltecimento dos tesouros florestais tropicais, assume caráter emblemático, como manifestação de civilização e progresso, aprovado e incentivado por empreendedores da época, intelectuais, políticos e tomadores de decisão (Candido, 2004; Perrotta, 2015; Capilé, França, Sales, 2021). Tal concepção alinhava-se, portanto, às práticas europeias já em curso desde os séculos XVI e XVII, as quais demandavam o deslocamento dos citadinos a fim de experienciar o frescor de outras áreas no verão e os benefícios para a saúde das estações minerais ou termais, assim como das regiões praianas (Perrotta, 2015).

Joaquim Manuel de Macedo (2005), médico e escritor do romantismo brasileiro, além de cronista do *Jornal do Commercio* entre 1862 e 1863, expressa tais valores em suas crônicas nesse diário, as quais foram reunidas no livro *Um passeio pela cidade do Rio de Janeiro*. Sua obra se caracteriza por romances urbanos e costumes burgueses, como apresentados, por exemplo, em *A moreninha* e o *O moço loiro*, para citar apenas dois (Bezerra, 2018). Descreve a cidade em transformação atento não só ao cenário material, mas também ao imaterial e sensível. Nessa condição de observador da cidade e de seus costumes num contexto global, Macedo expressa em um de seus artigos, em 1862, a censura ao comportamento dos cariocas que preferiam viajar à Europa a ocupar-se de conhecer sua própria cidade. Igual crítica se dá em artigos dos anos 1920, quando, na revista da Sociedade Brasileira de Turismo, destaca a monotonia da Europa em substituição a passeios maravilhosos que a cidade do Rio de Janeiro proporcionava (Perrotta, 2015).

Dom Pedro II desempenhou importante papel no melhoramento urbano da cidade, o que resultou em maior afluxo de estrangeiros que nela fixavam moradia. Disso resulta a produção dos primeiros guias “para estrangeiros” e “para visitantes”, mesmo que o viajante turista ainda fosse raro. O primeiro guia turístico brasileiro, em 1862, tem por título *Viagem pitoresca a Petrópolis para servir de roteiro aos viajantes e recordação deste ameno torrão brasileiro*. Nele são empregadas expressões tais como: morada da saúde e bem-estar, paraíso terrestre e Suíça eternamente verdejante, por exemplo. Somente em 1873 foi publicado o primeiro guia sobre o Rio de Janeiro, de autoria de Felix Ferreira, voltado para “indicar com clareza e precisão as repartições e estabelecimentos mais notáveis desta cidade”. Nesse mesmo ano, foi publicado, pela Editora Garnier, o primeiro livro sobre o Rio de Janeiro dedicado ao estrangeiro em estada na cidade, sem ilustrações ou mapas, intitulado *Guia do estrangeiro no Rio de Janeiro e uma notícia histórica sobre os principais monumentos* (Perrotta, 2015).

Visitar o exuberante e mais recente “jardim da cidade” passou a ser um desejo do público, porém, a área era de difícil acesso. Chegar ao maciço da Tijuca nas primeiras décadas do século XIX era possível somente a pé ou com o auxílio de equinos. Em meados dos Oitocentos, surge uma nova alternativa, e a área passa a ser acessível por bondes puxados a cavalos até a parte inferior da subida do Alto da Boa Vista; e, a parte mais alta, por carros de aluguel também puxados por equinos. No guia *Hand book of Rio de Janeiro*, publicado em 1887, observa-se um anúncio da empresa de carros Peres e Cia., especializada em oferecer visitas aos sublimes cenários montanhosos da cidade, referindo-se à localidade com as expressões Éden Fluminense e Passeios Pitorescos (Perrotta, 2015).

À vista disso, para o presente trabalho realizou-se uma pesquisa sistemática nos periódicos da época (1860-1900), utilizando o portal da Hemeroteca Digital da Biblioteca Nacional e as palavras-chave “Eden Fluminense” e “Passeios Pittorescos”. A escolha desses descritores justifica-se pela constante ocorrência de tais termos nos anúncios. O número de ocorrências para cada uma das expressões foi agrupado por décadas, enquanto as publicações foram analisadas, separadamente, para verificar quais tinham relação direta com o maciço da Tijuca (Tabela 1). No total, foram consultados 28 jornais e periódicos, dos quais 18 faziam menção aos passeios turísticos, com destaque para o *Jornal do Commercio* (RJ) e o *Almanak Laemmert: Administrativo, Mercantil e Industrial* (RJ), com maior volume de propagandas.

**Tabela 1:** : Número de ocorrências por décadas (1850-1899) para as palavras-chave “Eden Fluminense” e “Passeios Pittorescos”

Período	“Éden Fluminense”	“Passeios Pitorescos”
1850-1859	0	0
1860-1869	0	2
1870-1879	5	6
1880-1889	70	14
1890-1899	5	32

Fonte: elaborada pelos autores a partir de busca realizada na Hemeroteca Digital da Biblioteca Nacional.

Nos documentos analisados destacaram-se, principalmente a partir da década de 1870, as diversas propagandas de “roteiros turísticos” ao maciço da Tijuca, comercializados pelo empresário P.A.F. Peres, e usualmente intituladas Passeios Pitorescos e Piqueniques Restauradores. A intenção era promover um divertimento rústico, onde a paisagem florestal tropical poderia ser conhecida e contemplada. No entanto, essas atividades de lazer eram destinadas, sobretudo, à elite carioca, considerando os custos de sua realização.

Em 1873, um anúncio publicado no *Almanak Laemmert: Administrativo, Mercantil e Industrial* (RJ) (Figura 1) propagandeava passeios aprazíveis e refrigerantes pelo maciço da Tijuca, nos quais, a partir dos diferentes roteiros oferecidos, seria possível conhecer o “Círculo Pitoresco”, que permitiria um “grande passeio mágico”, possibilitando visitar, por exemplo, a “Vista Chinesa”, que seria um ponto para os “admiradores da natureza” (Figura 2), ou, ainda, a “Floresta Imperial”, onde se faziam “os viveiros e plantação das madeiras de lei mais notáveis do país” e a “Cascatinha Taunay”, onde “SS. MM. AA. Imperiais fazem os seus piqueniques” (Figura 3). Naquele ano, outras opções para o divertimento na natureza seriam a “Cachoeira Saudável”, a “Cascata Grande”, o “Castelo Tijuca”, o “Jardim Botânico” e a “Mesa do Imperador”. Dessa maneira, no total, eram oferecidas oito atrações. Percebe-se, ainda, como os elementos da natureza, tão característicos da cidade do Rio de Janeiro, ganhavam destaque, fossem pelas belas paisagens com suas diversificadas formas, contornos e cores para ser contempladas, ou cachoeiras e cursos d’água para se banhar e refrescar. O Jardim Botânico, por exemplo, nascido como um jardim de aclimatação, em 1808, propiciava ao visitante o contato com espécies da flora brasileira, assim como espécies exóticas (Bediaga, 2007), cujo conhecimento só poderia ser vivenciado a partir de viagens para fora do país.

**Figura 1:** : Anúncio destacando as oito atrações que poderiam ser visitadas no maciço da Tijuca (*Almanak Administrativo, Mercantil e Industrial do Rio de Janeiro*, 1873, p.456)

**Figura 2:** : Vista do Rio de Janeiro a partir da Vista Chinesa, foto de Marc Ferrez, c.1885 (Instituto Moreira Salles, Rio de Janeiro)

**Figura 3:** : Cascatinha da Tijuca, também conhecida como Cascatinha Taunay. Foto de Marc Ferrez, c.1885 (Instituto Moreira Salles, Rio de Janeiro)

A produção do ideal paradisíaco propiciado pode ser ilustrada pela imagem captada através das lentes de Ferrez (Figura 2), a qual valoriza a sinuosidade do relevo carioca, onde despontam o morro do Corcovado, sem o monumento do Cristo Redentor, e o Pão de Açúcar, assim como também o espelho d’água da Lagoa Rodrigo de Freitas, tomada a partir do sentimento de uma integração do observador com a floresta. Na reprodução da Cascatinha Taunay (Figura 3), a imagem exalta a grandiosidade da queda d’água e de toda a floresta circundante, sob o olhar de um visitante, numa possível provocação à reflexão sobre as escalas entre ser humano e natureza.

Em outro anúncio, publicado em 24 de janeiro de 1874, no periódico *A República*, observa-se a adição de novas atrações aos roteiros comercializados. A partir desse ano, quem adquirisse alguns dos pacotes poderia visitar outros pontos, como, por exemplo: “Alto do Archer”,^2^ que ofereceria a “vista dos botânicos”, “Boa Vista”, “onde existe o grande hotel Victoria”, “Cascatinha Freitas ou Grupo de Ninfas” e “Retiro do Ginty”. Eram, no total, 12 atrações.

No decorrer das décadas, percebe-se considerável acréscimo no número de atrações indicadas nos roteiros de Peres: de oito, em 1873, para 67, em 1890.^3^ Cada um desses pontos turísticos exibia qualidades próprias, razões específicas que lhes davam sentido e os tornavam dignos de ser visitados.

A ampliação da rede hoteleira, para além das regiões do Centro e do Catete, assim como a melhoria dos acessos, espraiando-se por Jardim Botânico, Laranjeiras, Gávea e Tijuca (hotéis Metrópole, Belvedere, Botanical Recreio de Olaria, Corcovado, Aurora etc.) facilitavam o consumo desse produto turístico que valorizava, por meio dos diferentes percursos, a natureza pujante da cidade do Rio de Janeiro (Perrotta, 2015).

Somente na publicação *Impressões de viagem Brasil-Europa*, de 1887, são apresentados os pontos de maior apelo turístico, entre os quais aparecem: o Corcovado, as Paineiras e o Alto da Boa Vista (incluídas a Cascata Grande, a Cascatinha Pequena e a Floresta Nacional da Tijuca). A eles estão associadas as estações de bonde e a disponibilidade de hotéis em suas proximidades (Perrotta, 2015). Nesse sentido, os bondes tiveram significativa importância para a expansão urbana na cidade, tendo como pioneira a Companhia de Carris de Ferro da Cidade à Boavista na Tijuca, cuja linha foi inaugurada, em 1859, pelo imperador (Von der Weid, 1994).

No início do século XIX, o contínuo e crescente fluxo de estrangeiros no Rio de Janeiro promoveu maior deslocamento entre as províncias, tendo a cidade como principal destino de visitantes que dispunham de tempo e recursos suficientes para conhecê-la e explorá-la (Machado, 2013), além dos equipamentos urbanos para facilitar sua visita.

Cabe destacar, ainda, que a procura pelos passeios para as áreas verdes do maciço da Tijuca também ocorria pela possibilidade de se experimentar um clima mais agradável. Tratava-se, então, da consagração de certos espaços higiênicos, em meio a uma cidade que crescia e precisava de áreas verdes, onde as condições de salubridade pudessem se consolidar (Cardozo, 2016).

## “Éden Fluminense”: transmutação do maciço da Tijuca na segunda metade do século XIX

A partir da análise dos anúncios de jornais de época foi possível identificar o valor simbólico atribuído ao maciço da Tijuca presente em plena malha urbana carioca, em meados dos Oitocentos. Esse resultado evoca um horizonte interessante em que a ênfase na totalidade seria uma das mais abrangentes dimensões constitutivas do movimento do romantismo (Duarte, 2005). Ao se aprofundar nesse elemento ideológico, entretanto, o autor enfatiza que a totalidade pode adquirir, comumente, o sentido de unidade, expresso, por exemplo, na consolidação do conceito moderno de nação, instituído ao longo do século XIX, por meio de árduo trabalho de incorporação sistemática dos tesouros, presentes em seu território, e dos hábitos e costumes de sua população, por intermédio da história. Nessa perspectiva, ganha destaque a busca por uma identidade nacional, esquadrinhada segundo um novo espírito de descrição, valorização e compreensão de suas tradições culturais e suas paisagens naturais.

Diferente das atitudes românticas que marcaram a construção da identidade de nações europeias, durante o século XIX, em que os costumes das populações rurais e as realizações materiais sucedidas por processos civilizatórios ao longo do tempo eram os principais elementos de autenticidade (Thiesse, 1999), no Brasil, descortinar e valorizar as matas locais representaram o caminho para a construção simbólica de uma pátria. Tanto a literatura como a poesia e a pintura se ajustavam ao desejo de diferenciação, retratando e elogiando as singularidades das riquezas das florestas brasileiras (Candido, 2004).

A vasta exploração dos trópicos e a descoberta de milhares de espécies que o mundo da ciência nunca tinha visto antes determinavam o surgimento de um novo paradigma estético na Europa e que reverberava para as demais regiões do globo: a valorização e apreciação de paisagens que encerrassem diversidade tão significativa de plantas quanto fosse possível, um espaço de contemplação da natureza e, ao mesmo tempo, útil ao conhecimento do ser humano acerca do mundo vegetal. Nessa perspectiva, ressaltam-se a importância das áreas verdes urbanas e seus usos sociais, tais como os “passeios pitorescos” e “piqueniques restauradores” no maciço da Tijuca. A instituição dos roteiros de visitas ao “Éden Fluminense”, cuidadosamente arquitetados, promovia o encantamento pela densa floresta tropical, sugerindo emoções por meio do encontro do belo com o sublime. Tais convicções seriam um ato de louvor às áreas verdes.

O desejo coletivo de experimentar os sentimentos de liberdade, paz, felicidade, alternados com sensações de tristeza e melancolia, simbolizavam uma fase de grande aproximação aos recursos naturais, por meio das constantes *promenades* nos jardins e parques metropolitanos. Estes se aprimoravam, cada vez mais, nos seus cenários sombrios, repletos de dramaticidade, na busca por reproduzir uma paisagem natural e intocada, estabelecendo os alicerces de uma nova relação entre o ser humano ocidental e a natureza (Schama, 1996).

Com a independência do Brasil, efetivada em 1822, a cidade do Rio de Janeiro tornou-se a sede do Império, figurando definitivamente seu papel de centro da nação e exercendo sua função fundamental de irradiação intelectual e artística (Candido, 2004). Nesse contexto, nada seria mais inteligente do que o enaltecimento das florestas e a proliferação dos recintos ajardinados, locais que sugeriam sofisticação e modernidade e para onde as famílias deveriam se dirigir para fazer piqueniques e apreciar as áreas verdes. Esses seriam espaços dos quais a metrópole brasileira poderia se orgulhar. Além de ser fortemente recomendados por sanitaristas para a recuperação da água do solo, insolação e circulação do ar, evitando a formação de sítios pútridos, favoráveis ao surgimento de moléstias (Lawrence, 2008), tão característicos de grande parte da cidade. Reverenciar, conhecer e reconhecer as particularidades da vegetação local representava uma manifestação de prosperidade para a capital tropical, sinalizando a tão desejada aproximação com as nações civilizadas europeias.

O presente estudo sugere, portanto, que o caráter contemplativo, diante de uma natureza nacional abundante e diversa, com inúmeras potencialidades, teve lugar de destaque no maciço da Tijuca. O número cada vez maior de atrações no interior da floresta, ao longo dos anos, manifestado nas propagandas dos jornais, pode ser lido sob a perspectiva de formação de uma memória da recente nação brasileira, como um artifício para tornar essas paisagens e locais verdes de lazer parte da imagem social e simbólica do país, constituindo, nesse sentido, uma atitude altamente federativa.

Segundo Duarte (2005), à medida que a natureza local se torna um atributo de orgulho e que esse sentimento patriótico se espalha e entranha na população, ele se configura numa peça de fundamental importância para o fortalecimento das cortes reais. O maciço da Tijuca experimenta essa ressignificação, passando a simbolizar para os habitantes do Brasil o glamouroso “Éden Fluminense”, um rico tesouro nacional, domesticado, acessível, fornecendo, dessa maneira, subsídios à concepção de uma consciência coletiva sobre a importância e a autenticidade de sua natureza, diferenciada das demais, adequando-se ao espírito de expressão de uma singular nação brasileira.

## Considerações finais

No contexto do maciço da Tijuca, as características pessoais dos três principais administradores da Floresta da Tijuca acabaram por nele imprimir suas personalidades, associadas às demandas sociais e políticas locais, bem como às tendências estrangeiras na relação de ser humano, sociedade, cidade e mundo natural. Nesse sentido, as atrações do maciço da Tijuca receberam os nomes desses personagens, por exemplo: Alto do Major Archer, Ponte do Barão d’Escragnolle e Refúgio Glaziou, para citar apenas três.

O pragmatismo de Archer em reflorestar e expressar a flora nativa que fora destruída é depreendido pelo uso preferencial de espécies naturais do Brasil no projeto dedicado, primeiramente, à proteção de nascentes e de cursos d’água. Optou também por selecionar, dentre essas espécies nativas, aquelas de vocação madeireira, numa perspectiva estratégica futura de geração de recursos financeiros para o Império. Assim, o manto florestal de que a cidade atualmente dispõe retrata a intencionalidade de Archer em seu desenho de projeto original, sem desconsiderar o concurso dos processos naturais de regeneração a partir das áreas plantadas.

A passagem de d’Escragnolle, homem culto, cujo olhar europeizado e sensível às artes se ocupou de embelezar a Floresta da Tijuca com a construção de caminhos, pontes, fontes e mirantes, consoante com os ideais românticos europeus de cidade, ampliou, assim, a dimensão desse espaço, concebido e compreendido como uma área de floresta protetora, para um parque público dedicado à visitação da população.

Glaziou, a despeito de sua breve passagem na administração, imprime também sua estética de botânico e paisagista da corte na coleta, produção, propagação e plantio de espécies vistosas de valor estético. Valeu-se também das espécies ocorrentes nas porções mais conservadas do maciço como fonte de espécimes não só para documentação em coleções de herbário, como também para uso paisagístico em distintas áreas na cidade.

Assim, as diferentes representações das paisagens artificiais, ao longo do tempo e no espaço, nos dão pistas que permitem penetrar a complexidade da relação das sociedades com o seu meio, bem como a transformação de um imaginário que irá conduzir à valorização de novos sentidos, e, quiçá, a uma nova geometria cognitiva da natureza.

Conhecer as diversas atrações no maciço da Tijuca, apresentadas em anúncios de jornais do século XIX, foi fundamental para compreender como as áreas verdes inseridas no contexto urbano carioca, juntamente com as paisagens naturais, expressavam as demandas, os interesses e as expectativas da sociedade brasileira, mais particularmente de uma elite aristocrática, que marcavam o período histórico em questão.

Contemplar, valorizar e vivenciar o maciço da Tijuca representavam, portanto, a construção de memória social federativa e de sentimento de afirmação de uma identidade brasileira que, a partir dos seus tesouros florestais e da exuberância da sua natureza, repleta de qualidades e potencialidades, se envaidecia de sua nação.

## NOTAS

1 De maneira sucinta, pode-se compreender “reflorestamento” como a reposição da cobertura florestal em áreas onde anteriormente existiram florestas (Magnanini, 1960). Considerando a complexidade e a extensa produção sobre tal temática, e que este artigo não se propõe a historiar o processo de reflorestamento do maciço da Tijuca nem discutir as políticas ambientais pretéritas sobre o assunto, recomenda-se a leitura de Rodrigues et al., (2009) para mais amplo entendimento sobre o tema desde o ponto de vista da restauração ecológica; ou de Moretto (2016) e Ribeiro e Moretto (2022) para melhor entendimento a partir da história ambiental, para citar poucos exemplos.

2 Trata-se de um topônimo que permanece até os dias atuais e é possível vê-lo a partir do Bom Retiro, no Setor Floresta da Tijuca, do Parque Nacional da Tijuca.

3 Em anúncio publicado no *Jornal do Commercio*, em 23 de dezembro de 1890, eram oferecidas as seguintes atrações: Caixa d’água Aurora, Caixa d’água Frias Vasconcellos, Avenida da Redemptora, Cascatinha Freitas, Praça da Boa Vista, Parque Itamaraty, Castello Leopoldina, Ermida Carvalhaes, Cachoeira Saudável (Posto da Comp. de Educação), Furnas de Agassiz, Grande Cascata Serpa Pinto, Círculo do Barão do Bom Retiro, Cascatinha do Barão de Taunay, Bica do Daniel, Parque Bonfim, Capella do Mesquita, Floresta Nacional, Praça Solon, Boulevard Deodoro, Rua Sampaio Ferraz, Ponte do Barão de Escragnolle, Caminho da Glória, Fonte Marianinha, Pedra Raul, Entrada do Paraíso, Praça Belford Roxo, Excelso, Rua Ruy Barbosa, Recreio Benjamin Constant, Rua Manoel Felizardo, Vista Wandenkolk, Fonte Jesuína, Éden Major Freitas, Alto do Major Archer, Rua Quintino Bocaiúva, Rua Major Campos, Rua Glicério, Praça d’Escragnolle, Praça Quinze de Novembro, Viveiro Florestal, Rua General José Simeão, Descanso Marietta, Cascata Diamantina, Ponte da Baronesa, Gruta de Paulo e Virgínia, Gruta do Constâncio, Abrigo do Chico Christão, Labirinto, Cascatinha Gabriella, Descanso de Dom Pedro de Alcântara, Lago das Fadas, Refúgio Glaziou, Rua Campos Salles, Rua Demétrio Ribeiro, Rua do Açude, Ponte do Chico da Serra, Retiro do Ginty, Parque Lemgruber, Pedra Bonita, Parque Cochrane, Castello Alencar, Recreio Mock, Vista dos Millords, Vista Chinesa e Jardim Botânico.
